# Comparison of myocardial protection methods in mitral valve surgery: a cohort study

**DOI:** 10.1186/1749-8090-10-S1-A171

**Published:** 2015-12-16

**Authors:** Eduardo AV Rocha, Lucas CD Pena, Rafael A Carvalho, Renato A Carvalho, Thiago A de Castro, Mateus D Freire

**Affiliations:** 1 Cardiovascular Surgery, Hospital Universitário Ciências Médicas, Belo Horizonte, Minas Gerais, 30140-073, Brazil; 2 Faculdade da Saúde e Ecologia Humana, Vespasiano, Minas Gerais, 33200-000, Brazil

## Background/Introduction

Myocardial protection is the group of strategies aimed at reducing ischemia-reperfusion lesions and its consequences. There is much discussion regarding different methods and their features, while there is no unanimity about which is most appropriate.

## Aims/Objectives

We aimed to compare two types of myocardial protection, intermittent global ischemia and cold crystalloid cardioplegia, in patients submitted to mitral valve surgery: preoperative conditions and surgical or clinical outcomes. Furthermore, we aimed to calculate cardioplegia exclusive equipments' price.

## Method

We performed a retrospective cohort study of medical records from patients who underwent mitral valve surgery at Hospital Universitário Ciências Médicas between 2010 and 2014. We compared preoperative conditions (gender, age and Euroscore II) and outcomes: death within 30 days, postoperative intensive care time and hospital stay time, cardiopulmonary bypass and ischemia time, use of pacemaker, use of vasoactive drugs and their time and intubation time. Cardioplegia equipments' total cost was calculated by indexation through three methods and thereafter the mean of its results.

## Results

We found no differences between the two groups' preoperative conditions. The only difference between outcomes were cardiopulmonary bypass and myocardial ischemia time, in which the intermittent global ischemia group was favored (Bypass: RR 0,19, 95% CI: 0,09 - 0,41; Ischemia: RR 0,20, 95% CI: 0,10 - 0,43).The mean price found for the cardioplegia equipment was R$321,03 (USD 101,12).

## Discussion/Conclusion

Intermittent global ischemia presented shorter surgical duration and reduced mitral valve surgery costs, while it didn't affect mortality or morbidity. More comparative studies including other surgery types and different services are necessary to validate our results.

